# Improved Anticancer Effect of Recombinant Protein izTRAIL Combined with Sorafenib and Peptide iRGD

**DOI:** 10.3390/ijms20030525

**Published:** 2019-01-27

**Authors:** Roman Fadeev, Alexey Chekanov, Marina Solovieva, Olga Bezborodova, Elena Nemtsova, Nadezda Dolgikh, Irina Fadeeva, Anatoly Senotov, Margarita Kobyakova, Yana Evstratova, Raisa Yakubovskaya, Vladimir Akatov

**Affiliations:** 1 Institute of Theoretical and Experimental Biophysics, Russian Academy of Sciences, Pushchino, Moscow Region 142290, Russia; fadeevrs@gmail.com (R.F.); alex256@yandex.ru (A.C.); ms0@mail.ru (M.S.); n.v.dolgikh@gmail.com (N.D.); a.s.senotov@gmail.com (A.S.); ritaaaaa49@gmail.com (M.K.); yannaevstratova@gmail.com (Y.E.); 2 P. Hertzen Moscow Oncology Research Institute, Ministry of Health, Moscow 125284, Russia; olgabezborodova@yandex.ru (O.B.); nemtz@yandex.ru (E.N.); raisayakub@yandex.ru (R.Y.); 3 Pushchino State Institute of Natural Sciences, Ministry of Education, Pushchino, Moscow Region 142290, Russia

**Keywords:** TRAIL/Apo2L, multicellular TRAIL-resistance, sorafenib, tumor-penetrating peptide iRGD, HT-1080 human fibrosarcoma cells

## Abstract

One of the main problems in oncology is the development of drugs that cause the death of cancer cells without damaging normal cells. Another key problem to be solved is to suppress the drug resistance of cancer cells. The third important issue is to provide effective penetration of drug molecules to cancer cells. TRAIL (TNFα-related apoptosis inducing ligand)/Apo2L is a highly selective anticancer agent. However, the recombinant TRAIL protein having high efficiency against cancer cells in vitro was not effective in clinical trials. Recently we have discovered an acquisition of TRAIL resistance by cancer cells in confluent cultures, which is apparently a manifestation of the general phenomenon of multicellular resistance. The aim of this study was to evaluate whether the anticancer effect of the recombinant protein TRAIL in vivo can be improved by the suppression of multicellular TRAIL-resistance using sorafenib and a tumor-penetrating peptide iRGD, c(CRGDKGPDC). The results testified a great increase in the resistance of human fibrosarcoma HT-1080 cells to izTRAIL both in confluent cultures and in spheroids. Sorafenib administered at nontoxic concentration effectively suppressed confluent- or spheroid-mediated TRAIL-resistance of HT-1080 cells in vitro. Sorafenib combined with iRGD significantly improved the anticancer effect of the recombinant protein izTRAIL in HT-1080 human fibrosarcoma grafts in BALB/c nude mice. Consistent with this finding, multicellular TRAIL-resistance may be a reason of inefficacy of izTRAIL alone in vivo. The anticancer effect of the recombinant protein izTRAIL in vivo may be improved in combination with sorafenib, an inhibitor of multicellular TRAIL resistance and iRGD, the tumor-penetrating peptide.

## 1. Introduction

One of the main problems of oncology is the creation of drugs that cause the death of cancer cells with no damage to the cells of healthy tissues [1]. Another key problem is to suppress the drug resistance of cancer cells [2]. The third important issue is to improve the penetration of drug molecules to cancer cells [3]. The particular interest for development of highly selective anticancer drugs is related to the cytokine TRAIL (TNFα related apoptosis inducing ligand).

TRAIL is an integral membrane protein overexpressed in some subpopulations of natural killer and dendritic cells. TRAIL binds to DR4 and DR5 receptors on the cell surface and induces apoptosis of cancer cells without damaging normal cells [4,5,6]. The results obtained in vitro have shown that some tumor cells are resistant to TRAIL [7,8]. It was recently shown that TRAIL-sensitive cancer cells derived from human carcinomas of different origin can acquire the resistance to TRAIL-induced apoptosis in confluent cultures and spheroids [9,10]. It was found that confluence-mediated TRAIL resistance may be suppressed by some target drugs, for instance by sorafenib, added at nontoxic concentrations [11]. These results indicate the opportunity to overcome multicellular TRAIL resistance of cancer cells using target drugs.

To solve the third problem, the limitation of drug transfer to cancer cells, the tumor-penetrating peptide iRGD has been proposed recently [12,13]. This peptide binds specifically to αVβ3 and αVβ5 integrins of the endothelium in tumors, and after proteolytic conversion occurring on the endothelial cell surface the resulted peptide detaches from integrins and binds with receptor neuropilin 1, improving transendothelial permeability of blood vessels and drug transfer into tumor cells [13]. In our work the attempt was made to evaluate the necessity to solve all the three abovementioned problems to improve the efficiency of the antitumor recombinant protein izTRAIL.

## 2. Results

### 2.1. Increase in Resistance of Cancer Cells HT-1080 to izTRAIL in Confluent Cultures

Our study demonstrated that the toxic effects of izTRAIL were different significantly in the nonconfluent and confluent cultures of human fibrosarcoma cells HT-1080.

**Figure 1:**
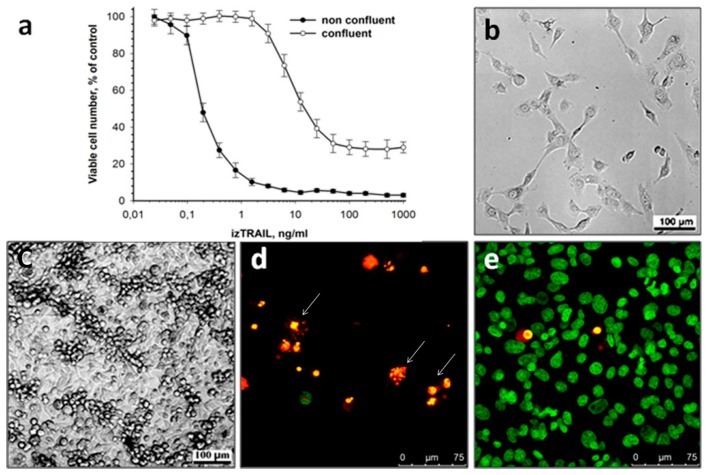
Increase in the resistance of HT-1080 cells to the recombinant protein izTRAIL in a confluent culture. (**a**) Cell viability vs. izTRAIL concentration in nonconfluent (24 h after seeding) and confluent (96 h after seeding) cultures, *n* = 5; (**b**,**c**) representative images of nonconfluent and confluent cultures, correspondingly; (**d**,**e**) representative images of nonconfluent and confluent cultures, correspondingly, in one day after the addition of 1.5 ng/mL of izTRAIL. The cultures were stained with cell nuclear dyes H33342 and PI, 1 µg/mL each. Green nuclei—viable cells, yellow-red nuclei—dead cells. Aberrant allocation of chromatin (arrow) indicates apoptosis.

To evaluate the density-dependent toxic effect of the recombinant protein izTRAIL, cell cultures were treated with izTRAIL in 1 day (nonconfluent cultures) and 4 days (confluent cultures) after seeding of 10^4^ cells per well. The cells were sensitive to the protein izTRAIL one day after the seeding, and the concentration of izTRAIL, IC50, at which the number of viable cells decreased to 50% relative to the control (nontreated cultures), was of 0.2 ± 0.05 ng/mL (Figure. 1). Four days after cell seeding, the cultures looked as a confluent monolayer of spread cells with some aggregates of spherical cells lying on the monolayer. The sensitivity of cells to izTRAIL in confluent cultures reduced relative to nonconfluent cultures. This increase in TRAIL resistance manifested in the fact that about 30% of confluent culture cells become insensitive to izTRAIL (plateau level at high concentrations of izTRAIL), and the IC50 value for a subpopulation of cells remaining sensitive to izTRAIL (determined by the percentage of living cells from 100% to a plateau in Figure 1a) was of 10 ± 2 ng/mL. The micrographs of nonconfluent (TRAIL-sensitive) and confluent (TRAIL-resistant) cultures of HT-1080 cells are presented in Figure 1b,c, correspondingly. Panels d and e in Figure 1 show cell viability in nonconfluent and confluent cultures treated with 1.5 ng/mL of izTRAIL. It is seen that in accordance with the data presented in Figure 1a, this treatment is highly effective in nonconfluent cultures and ineffective in confluent cells. Therefore, our results show a significant increase in TRAIL resistance of HT-1080 human fibrosarcoma cells in confluent cultures.

### 2.2. Suppression of Confluence-Mediated TRAIL Resistance of HT-1080 Cells by Sorafenib

**Figure 2:**
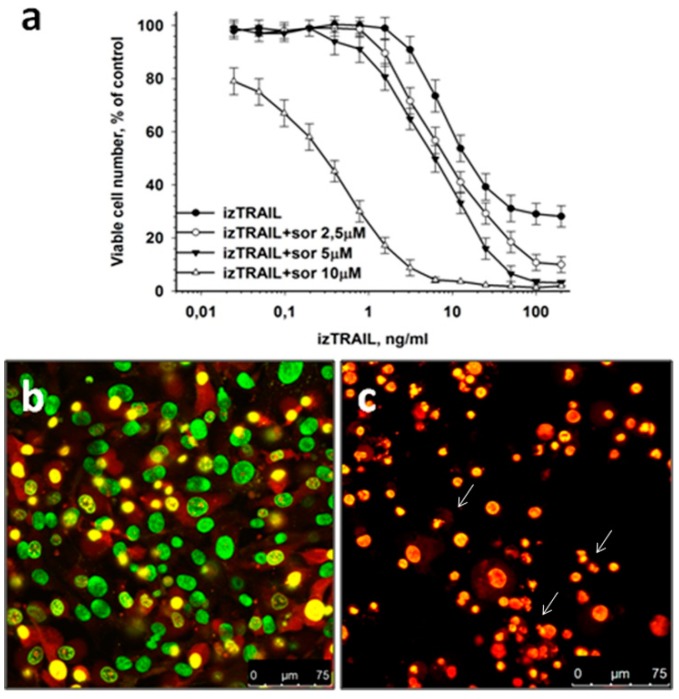
Suppression of confluent izTRAIL resistance by 10 μM sorafenib. (**a**) Cell viability vs. concentration of izTRAIL in confluent (96 h after seeding) cultures in one day after the addition of izTRAIL and sorafenib, *n* = 5; (**b**,**c**) representative images of confluent cultures in one day after the administration of 10 μM sorafenib and a combination of 10 μM sorafenib and 1.5 ng/mL izTRAIL, respectively. The cultures were stained with nuclear dyes H33342 and PI, 1 mkg/mL each. Green nuclei—viable cells, yellow-red nuclei—dead cells. Aberrant allocation of chromatin (arrow) indicates apoptosis.

It was shown previously that the resistance of some carcinoma cells to TRAIL-induced apoptosis acquired in confluent cultures can be suppressed by sorafenib added at a nontoxic concentration [11]. In the present article we evaluated the effect of sorafenib, an inhibitor of several tyrosine protein kinases (VEGFR, PDGFR) and Raf kinases, on the confluence-dependent TRAIL resistance of human fibrosarcoma HT-1080 cells. We demonstrated that sorafenib, added at nontoxic concentrations of 2.5 and 5 μM, together with izTRAIL reduced the percentage of TRAIL resistance in HT-1080 cells from 30% to 10% and 0%, respectively (plateau at high concentrations of izTRAIL in Figure 2a). Sorafenib in a concentration of 10 μM had low toxic effect when applied alone and fully suppressed the confluence-dependent TRAIL resistance when combined with protein izTRAIL, reducing the value of IC50 to 0.4 ± 0.1 ng/mL (Figure 2a). The fluorescence micrographs in Figure 2 illustrate the resistance of confluent HT-1080 cells to 10 μM of sorafenib (Figure 2b), similar to that against 5 ng/mL of izTRAIL, and the total apoptotic cell death induced by a combination of sorafenib (10 μM) and izTRAIL (1.5 ng/mL) (Figure 2c).

Thus, sorafenib applied in nontoxic concentrations effectively suppressed TRAIL resistance of human fibrosarcoma HT-1080 cells, which is acquired in confluent cultures.

### 2.3. TRAIL Resistance of HT-1080 Cells Acquired in Spheroids

**Figure 3:**
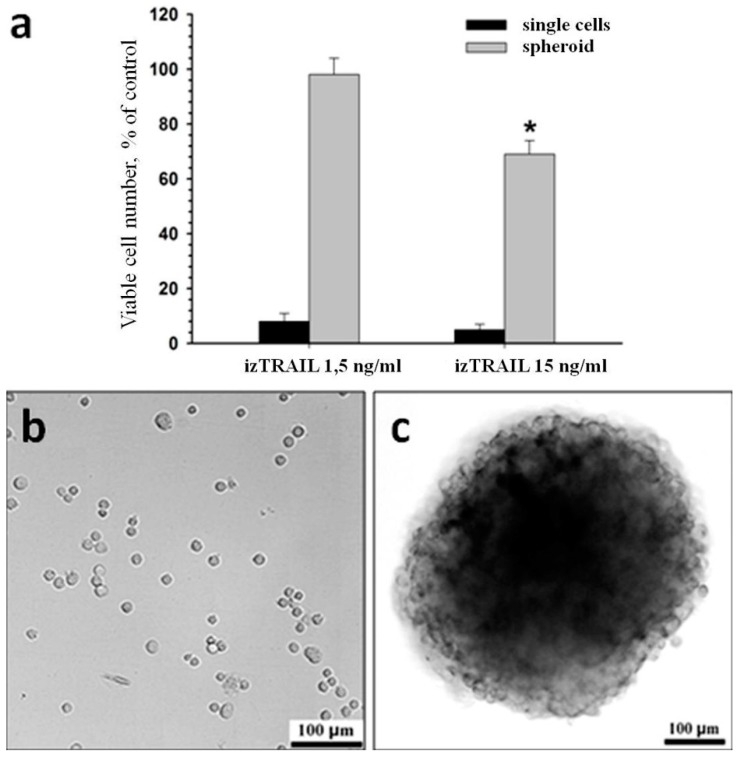
Increase in izTRAIL resistance of HT-1080 cells in spheroids. (**a**) Cell viability in spheroids or cell culture one day after the addition of izTRAIL immediately after seeding, *n* = 5. (**b**) representative images of cell culture after seeding; (**c**) a typical spheroid. * *p* < 0.05.

To evaluate the potential TRAIL resistance of tumor HT-1080 cells *in vivo*, the effect of izTRAIL on spheroids and single non-spread cells was compared. TRAIL sensitivity of spherical cells immediately after seeding, and spread cells in 24 h after seeding, were equal. In particular, the survival rate was ≤5% at izTRAIL concentrations higher than 2 ng/mL, and the IC50 value of izTRAIL was of 0.2 ± 0.05 ng/mL. The TRAIL sensitivity of cells in spheroids was significantly lower relative to single cells. For example, at izTRAIL concentration of 1.5 ng/mL single spherical cells were mostly dead, while cells in spheroids remained viable (Figure 3a). This difference persisted at TRAIL concentration of 15 ng/mL. Figure 3b,c show microphotographs of spheroids and spherical single cells in these experiments.

**Figure 4:**
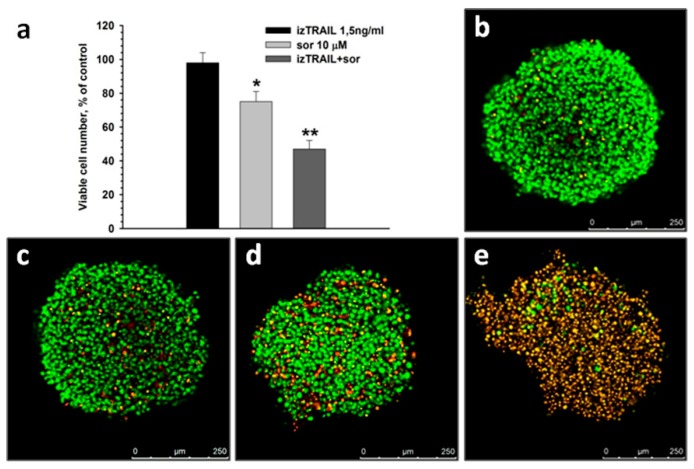
Suppression of TRAIL resistance of HT-1080 cells in spheroids by 10 μM sorafenib (**a**). (**b**–**e**) Confocal microscopy of the spheroids (merge of z-stacks). The cultures were stained with nuclear dyes H33342 and PI, 1 µg/mL each. Green nuclei—viable cells, yellow-red nuclei—dead cells. (**b**) control, without treatment; (**c**) in one day after the addition of 1.5 ng/mL izTRAIL; (**d**) in one day after the addition of 10 μM sorafenib; (**e**) in one day after the addition of 1.5 ng/mL izTRAIL and 10 μM sorafenib. *n* = 5, * *p* < 0.05, ** *p* < 0.01.

Sorafenib suppresses the multicellular TRAIL resistance of HT-1080 cells in spheroids. For example, izTRAIL at a concentration of 1.5 ng/mL decreased the percentage of living cells in spheroids to 55 ± 6% when combined with 10 μM of sorafenib, while 1.5 ng/mL of izTRAIL alone was nontoxic and 10 μM of sorafenib alone lowered the percent of live cells to merely 75 ± 6% (Figure 4). This result indicates a synergistic action of izTRAIL combined with sorafenib. Synergism of izTRAIL and sorafenib was less pronounced in spheroids than in confluent cultures (Figure 2a and Figure 4a). However, in a layer of about 50 µm near the spheroid surface the total cell death was observed after administration of 1.5 ng/mL izTRAIL in combination with 10 μM of sorafenib (Figure 4e) in contrast to only partial cell death throughout the spheroids (Figure 4a). This difference points at the surviving cells inside spheroids during the treatment with the combination of 1.5 ng/mL izTRAIL and 10 μM of sorafenib.

### 2.4. Improvement of Anticancer Effect of the Recombinant Protein izTRAIL Combined with Sorafenib and the Peptide iRGD

To understand the effect of the peptide iRGD *in vivo*, a combined action of iRGD, izTRAIL and sorafenib on fibrosarcoma HT-1080 cells was studied in vitro with the peptide concentration of 4 μg/mL, which was effective for the improvement of trans-endothelial mass transfer in tumors according to E. Ruoslahti and coworkers [12,13]. It was revealed that the peptide iRGD added at concentrations of up to 100 μg/mL had no toxic effect on HT-1080 cells in both confluent and nonconfluent cultures (data not shown). The peptide iRGD did not influence on the effects of izTRAIL, sorafenib, and their combination in HT-1080 cells in confluent and nonconfluent cultures, as well as in spheroids.

The multicellular resistance of cancer cells to TRAIL-induced apoptosis may be expected to inhibit significantly the effectiveness of the antitumor effect of the recombinant TRAIL protein. Therefore we evaluated whether sorafenib improves the suppression of tumor growth by izTRAIL in mice, and whether the peptide iRGD improves the antitumor effect from the concomitant use of izTRAIL and sorafenib.

**Figure 5:**
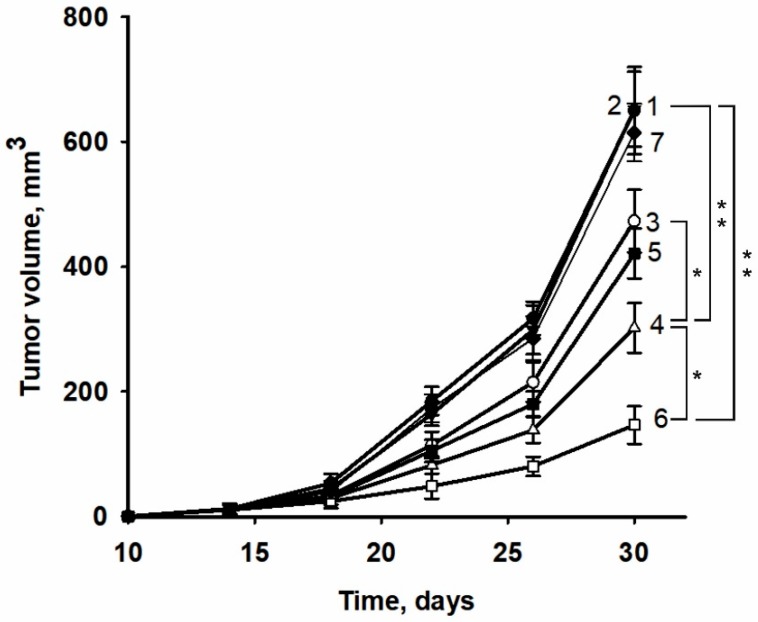
Tumor growth induced by an injection of HT-1080 human fibrosarcoma cells in control (1), treated according to the experimental protocol (see Materials and Methods section) by izTRAIL (2), sorafenib (3), izTRAIL and sorafenib (4), sorafenib and iRGD (5), izTRAIL and sorafenib and iRGD (6), izTRAIL and iRGD (7) * *p* < 0.05, ** *p* < 0.01.

It was found that the recombinant protein izTRAIL applied alone did not suppress the growth of HT-1080 tumors (Figure 5, curves 1 and 2), in contrast to the high efficiency of the apoptosis-inducing activities of these proteins in low density cultures of HT-1080 cells.

Sorafenib administered alone provided a weak suppression of tumor growth (Figure 5, curve 3). When combined with izTRAIL sorafenib caused a more pronounced inhibition of tumor growth than sorafenib alone (Figure 5, curve 4) that may be due to inhibition of main pathways of cell survival such as Akt, MAPK. The peptide iRGD did not enhance the antitumor effect of sorafenib (Figure 5, curve 5). The combination of iRGD treatment with the administration of izTRAIL and sorafenib resulted in a significant increase in the inhibition of tumor growth (Figure 5, curve 6).

Thus, izTRAIL alone was not effective *in vivo*, whereas it inhibited tumor growth in a synergistic manner in a combination with sorafenib. Co-administration of iRGD together with izTRAIL and sorafenib synergistically enhanced the antitumor activity of the combination.

## 3. Discussion

Previously it was found that cell lines derived from various human carcinomas, including carcinomas of skin (A431), larynx (HEp-2), ovarian (OVCAR-3), and other types, acquire resistance to TRAIL-induced apoptosis in three-dimensional spheroids and two-dimensional confluent cultures [9,10,11]. In this study we demonstrated that confluent resistance took place not only in carcinoma cells, but also in the cells of fibrosarcoma. According to our data (Figure 1), the sensitivity of cells to TRAIL-induced apoptosis in confluent HT-1080 cell cultures decreased with the appearance of a subpopulation insensitive to izTRAIL (plateau level at high concentrations of izTRAIL in Figure 1). The mechanism of confluent resistance of tumor cells against TRAIL-induced apoptosis remains unknown. We consider the confluent resistance as an ability of tumor cells occurring in multicellular structures to acquire resistance to damaging impacts (multicellular resistance), which most likely take place in tumors. For this reason, the study of confluent resistance is not only of scientific interest but also has high practical significance.

In our study we revealed the suppression of confluent TRAIL resistance of HT-1080 fibrosarcoma cells by means of sorafenib, an inhibitor of some tyrosine kinase receptors (PDGFR, VEGFR) and Raf kinases [14]. Thus, sorafenib added at a nontoxic/subtoxic concentration was capable to transform a TRAIL-resistant cell subpopulation to a TRAIL-sensitive characteristic for nonconfluent cultures. It was revealed in our experiments that 50% inhibition of cell viability in HT 1080 confluent cultures for sorafenib was equal to 29 ± 4 μM. Therefore, sorafenib (10 μM) and izTRAIL (0.2 ng/mL) had synergistic toxic effect for confluent cultures with a Chou-Talalay combination index (CI) of 0.35 for 50% inhibition of cell viability. The effect of sorafenib may be due to inhibition of kinases of survival pathways.

The increase in TRAIL-resistance of HT-1080 cells in multicellular structures was confirmed in another well-known model—in spheroids. However, the suppression of multicellular TRAIL resistance by sorafenib in HT-1080 spheroid cells was less pronounced than in confluent cultures, and most clearly appeared on the surface of the spheroids. The difference in the effect of izTRAIL combined with sorafenib in these models may be due to the induction of some additional mechanisms of TRAIL resistance in spheroids or because of the possible limitations of mass transfer into spheroids. Thus, the confluent cultures can be considered as a suitable model for study of the mechanisms of the multicellular TRAIL resistance of tumor cells, whilst spheroids presumably reflect not only an increase in cell resistance themselves, but also the problem of drug transfer into tumor cells. An increase in the resistance of A549 cells against a combination of bortezomib and TRAIL in spheroids in comparison to monolayer culture has been reported earlier [10]. A similar effect was obtained for human mesothelioma cells subjected to a combination of TRAIL with cycloheximide [15]; however, the confluent TRAIL resistance or spheroid-mediated resistance of cancer cells against TRAIL alone were not demonstrated.

A significant result of our work is the fact that izTRAIL, effectively causing the death of HT-1080 cells in vitro, was completely ineffective as a monotherapy in vivo. Our results allow proposing the multicellular TRAIL resistance as a reason for inefficiency of the application of izTRAIL alone in vivo. This assumption was confirmed by the fact that sorafenib effectively inhibited the multicellular TRAIL resistance of HT-1080 cells in confluent cultures and multicellular spheroid in vitro, and improved the antitumor effect of izTRAIL in the HT-1080 tumor model in immunodeficient mice in a synergistic manner.

The limitation of drug transfer to tumor cells is one of the key problems of oncology [16,17]. To improve the transfer efficiency several penetrating peptides have been proposed recently, in particular, a cyclic peptide iRGD [12]. It was assumed that the iRGD peptide binds to the αvβ3 and αvβ5 integrins, which are overexpressed on endothelial cells in cancers. The iRGD peptide bonded on cell surface is subjected to proteolysis and CRGDK, resulting in the formation of a truncated iRGD peptide form with an exposed CendR motif. The CendR motif (R/KXXR/K) provided high affinity of CRGDK to neuropilin-1 leading to an increase in drug penetration through the endothelium to the cancer cells [12,13,18]. The iRGD peptide was shown to induce an increase in the penetration of doxorubicin and therapeutic antibody trastuzumab in the tumor [12] and of HPRP-A1 peptide in A549 3-D cellular spheres in vitro and in xenografts in vivo [19]. Furthermore, an enhancement of the antitumor effect of trastuzumab, due to its combination with iRGD, has been demonstrated in a model of BT-474 human breast carcinoma in mice [12]. In our work, the addition of the peptide iRGD significantly increased the inhibition of growth of HT-1080 human fibrosarcoma xenografts in nude mice caused by a combination of izTRAIL with sorafenib while dual therapy with izTRAIL and iRGD had no antitumor effect.

Thus the anticancer protein izTRAIL, being effective in vitro, was not effective in vivo without solving the problems of drug resistance and the improvement of drug transfer to cancer cells. Sorafenib, an inhibitor of multicellular TRAIL resistance, combined with the penetrating peptide iRGD significantly enhanced the anticancer effect of the recombinant protein izTRAIL in HT-1080 human fibrosarcoma grafts in BALB/c nude mice. The presented results demonstrate the necessity of an integrated solution of all the mentioned key problems of oncology for effective anticancer therapy.

## 4. Materials and Methods

### 4.1. Protocol of izTRAIL Preparation

The sequence of the synthetic isoleucine zipper (IEKKIEA)_4_ (RMKQIEDKIEEILSKIYHIENEIARIKKLIGE) [20] was synthesized de novo. The synthesis of the izTRAIL gene was performed as described earlier [21]. The resulting gene was cloned into the plasmid vector pET101 (Novagen, Madison, WI, USA). The *E. coli* strain BL21 (DE3) was transformed with the resulting expression vector pET101/izTRAIL. The expression of the protein was carried out in TB medium for 12 h at 18 °C. Cell biomass extraction was performed in a buffer solution containing 0.1 M Tris, 0.2 M NaCl, 50 mM EDTA (pH 8.0). Protein izTRAIL was mainly (90%) contained in inclusion bodies. Renaturation of the recombinant protein was performed in 6M guanidine hydrochloride solution, followed by the transfer of the protein to a buffer solution containing 0.5 M arginine, 0.5 M sodium chloride, 100 mM Tris-hydrochloride (pH 8.0) and 2 mM DTT to a final concentration of 500 µg/mL. The resulting izTRAIL (trimeric form is about 80 kDa, monomeric form is 27 kDa) was purified by metal ion affinity chromatography using Ni Sepharose High Performance as a sorbent. The purity of the protein was assessed using SDS-PAGE and Coomassie Brilliant Blue staining.

### 4.2. Synthesis of iRGD Peptide

The cyclic peptide iRGD, c(CRGDKGPDC), was synthesized by solid-phase chemical method in the Institute of Bioorganic Chemistry of RAS (Moscow, Russia). The peptide was prepared using Boc-protection of α-amino groups in amino acids [22,23]. The cleavage of the peptide from the resin and the concurrent removal of the permanent protecting groups were carried out using liquid hydrogen fluoride in a single step as described in [24]. The linear peptide was dissolved in a 0.1 M degassed solution of ammonium bicarbonate and stirred slightly with free access laminar air flow for 5 h for peptide cyclization.

The solution was lyophilized, the residue was dissolved in glacial acetic acid and the acetate form of the peptide was precipitated with ether. The homogeneity of the peptide (90–95%) was estimated by analytical HPLC (Column Nucleosil 120-5C18, 4.6 × 250 mm; Macherey-Nagel, Munich, Germany) and ESI-MS mass spectrometry: 948.6 [M + H]^+^; 475.4 [M +2 H ]^2+^, the theoretical values are 949.07/475.37, respectively.

### 4.3. Cell Culture

The human fibrosarcoma cell line HT-1080 was obtained from the Russian Cell Cultures Collection (Institute of Cytology, Russian Academy of Sciences, St. Petersburg, Russia). Cells were grown in DMEM culture medium (Sigma) supplemented with 10% fetal calf serum (Gibco) and 40 mg/L gentamicin at 37 °C in a humidified atmosphere of 5% CO_2_.

### 4.4. Formation of Spheroids

For studying the multicellular resistance of tumor cells to TRAIL-induced apoptosis in vitro, spheroids were used. Formation of multicellular spheroids was performed in 96-well Nunc culture plates (Thermo Scientific, Waltham, MA, USA) coated with 1.5% agarose [25]. After plating 5 × 10^3^ cells in 200 µL of growth medium per well the cultures were incubated for 96 h at 37 °C in a humidified atmosphere of 5% CO_2_.

### 4.5. Cytotoxicity Assay

For the cytotoxicity assay, cells were seeded in 96-well Nunc culture plates (Thermo Scientific) (10^4^ cells in 100 µl of growth medium per well). The cytotoxicity was evaluated using the crystal violet assay evaluating the ratio of optical densities at 560 nm in the treated and untreated (control) cultures at 24 h after the addition of substances [26]. The optical density value was in direct proportion to the number of viable cells. The number of viable cells was estimated by the trypan blue exclusion assay after cell culture trypsinization. To assay cell viability in spheroids the aggregates were treated with trypsin (0.25%)/EDTA (0.02%) solution in PBS to suspend the cells, and the live and dead cells were subsequently counted using trypan blue exclusion assay.

### 4.6. Assessment of Apoptotic and Mitotic Cells by Fluorescence Microscopy

The cultures were stained simultaneously with fluorescent DNA-binding dyes Hoechst 33342 (H33342, 1 μg/mL) and propidium iodide (PI, 1 μg/mL) to evaluate cell viability (staining by H33342 only) and to determine mitosis (mitotic chromatin allocation) and the type of cell death (necrosis or apoptosis). Cells with condensed or fragmented chromatin or with marginal allocation of chromatin were counted as apoptotic [27].

The analysis of cell viability in spheroids was performed using confocal microscopy and simultaneous staining with H33342 and PI. The confocal microscope permits to observe cells at a distance up to 50–60 µm from the surface of a spheroid. The merges of z-stacks are presented in the paper allowing to evaluate cell viability in the 50 µm layer near the spheroid surface.

### 4.7. Cancer Xenograft Model and Protocols of Anticancer Treatment

The model of human cancer grafts in immunodeficient mice was used to study the anticancer effect of izTRAIL combined with sorafenib (Sigma) and the iRGD peptide. Male BALB/c nude mice weighing 20–22 g, were obtained from the Laboratory Animal Breeding Facility “Pushchino” (Pushchino Branch of the Shemyakin and Ovchinnikov Institute of Bioorganic Chemistry of Russian Academy of Sciences).

One million HT-1080 cells suspended in 0.3 mL of PBS were injected subcutaneously in the side of each mouse. After 7 days following cell injection the animals were randomly divided into 6 groups listed below and treated daily for 21 days. PBS, izTRAIL and iRGD solutions in PBS were injected intravenously in mice tails. Sorafenib was administered orally. The mice were treated with PBS (0.3 mL) in the control group (1), with izTRAIL in a dose of 15 mg/kg in 0.3 mL PBS in group 2, with sorafenib (Nexavar, Roche) in a dose of 50 mg/kg in group 3, with izTRAIL in a dose of 15 mg/kg in 1 h after sorafenib administration (50 mg/kg) in group 4, with peptide iRGD in a dose of 4 mg/kg in 1 h after sorafenib administration (50 mg/kg) in group 5, and with peptide iRGD (4 mg/kg) together with izTRAIL (15 mg/kg) in 1 h after sorafenib administration (50 mg/kg) in group 6. Each group included seven mice. The tumor volume (V) was evaluated using the formula V = a*b^2^/2 [28]. The parameters a and b are the length and width of a tumor measured with a caliper. All experiments were performed in accordance with the “Regulations for Studies with Experimental Animals” (Decree of the Russian Ministry of Health of 12 August 1997, No. 755). The protocol was approved by the Commission on Biological Safety and Ethics of the Institute of Theoretical and Experimental Biophysics, Russian Academy of Science (November 2014, protocol N45).

### 4.8. Statistical Analysis

The results are presented as mean ± standard error (M ± SEM). Each of the in vitro experiments was carried out at least five times (*n* ≥ 5). The statistical significance of the results was analyzed using the Wilcoxon–Mann–Whitney test (*p* < 0.05).

## 5. Conclusions

Thus, the anticancer protein izTRAIL, being effective in non-confluent cell culture was not effective in spheroids and in confluent HT-1080 cells in vitro as well as in vivo in HT-1080 human fibrosarcoma grafts in nude mice. Sensitization of these cells to TRAIL-induced apoptosis with sorafenib increases TRAIL cytotoxicity significantly in vitro and slightly in vivo. Combination of sorafenib, an inhibitor of multicellular TRAIL resistance, with the penetrating peptide iRGD significantly enhanced the anticancer effect of the recombinant protein izTRAIL in HT-1080 human fibrosarcoma grafts in nude mice. The presented results demonstrate the necessity of an integrated solution of all the key problems of oncology for effective anticancer therapy including drug selectivity, drug resistance and drug penetration.
